# Club Foot: Its Nature and Treatment

**Published:** 1895-03-09

**Authors:** Walter L. Woollcombe

**Affiliations:** Assistant Surgeon South Devon and East Cornwall Hospital


### Medical Progress and Hospital Clinics.

[*The Editor will be glad to receive offers of co-operation and contributions from members of the profession. All letter should be addressed to* The Editor, The Lodge, Porchester Square, London, W.]

### CLUB-FOOT: ITS NATURE AND TREATMENT.

By Walter L. Woollcombe, F.R.C.S.E., Assistant Surgeon South Devon and East Cornwall Hospital.

Club-foot is a condition which is frequently brought to the notice of every medical practitioner as well as to those who make orthopædic surgery a special study, and, of course, in its congenital form the general practitioner is the first to be consulted, and has the advantage of seeing the deformity at a time when it can most profitably be taken in hand. For these and other reasons it is a subject which from time to time it is highly desirable to bring forward. I propose to consider the subject under the heads: (1) Mode of origin. (2) Different forms. (3) Treatment. And, first, as to mode of origin. In considering this part of the subject we must divide our cases into two large groups—the congenital and the acquired, although it is doubtful if even these can be arbitrarily divided; there are cases on the borderland, and I shall hope to show that many, in fact most, cases of both groups have a similar origin. On the whole, however, it will be more convenient to consider the two classes separately.

**Congenital Club-foot.**—The congenital cases are naturally those round which the fiercest controversy has raged, as the determination of their mode of origin must be to a certain extent a matter of conjecture, from the impossibility of making observations at the time the deformity commences. The number of explanations that have been put forward show a great deal of ingenuity in the medical mind, but are somewhat confusing to the seeker after truth. The more important are: (1) That the position of the fœtus in utero is the exciting cause; this view was upheld by Cruveilhier and Boyer. (2) That the primary cause is malposition of the tarsal bones. Scarpa and, at one time, Delpech both supported this theory. (3) Abnormal muscular action. Believed in by some German writers. (4) Disordered nervous action, including nerve storms, producing clonic or tonic spasm of certain muscles, or paralysis. This theory was first introduced by Rudolph, and afterwards strongly supported by Jules Guérin. (5) That some forms, notably talipes varus, are grades in the natural development of the feet. This theory, which was held by Von Walther, would only account for certain forms, and could not apply to talipes equinus or valgus. (6) Yet another theory was started by Eschricht, which depended on the fact (which he claims to have established) that in the early stages of intra-uterine existence, the lower extremities lie with their posterior surfaces against the abdomen, and that in the early months of gestation they rotate on their axes. If this rotation is not fully accomplished, club-foot results, and if the rotation does not take place at all the legs become united, and a condition resembling the "Siren" is the result. (7) Perhaps, as a last theory, "maternal impressions" should be mentioned.

We will now criticise these theories in detail in the order in which I have placed them.

First: That the position of the fœtus in utero is the exciting cause. The originators of this theory meant, of course, that the cramped position of the fœtus in utero led to so much pressure on the doubled-up feet, that the muscles and ligaments were permanently shortened and the feet unable, when released, to assume any other position. This theory must be rejected for the following reasons: (*a*) There never is any such pressure, for, as early as the fourth month, the quantity of liq. amnii is two and a half ounces, or half the bulk of the embryo, quite sufficient to prevent any unequal pressure on the limbs. In fact, if there was not sufficient fluid present to cause an even distribution of pressure the circulation would be so interfered with that the child could never be born alive. (*b*) If pressure on a part that was originally well formed were the cause the child born with club-foot ought also to exhibit contractions of the knee, hip, arms, and neck, which are equally exposed to pressure, but such is not the case. (*c*) That in many cases of deficient liquor amnii which have been reported no mention is made of club-foot, and, on the other hand, many cases of club-foot are reported where there was excess of the fluid.

In support of the second theory, "that the primary cause is malposition of the tarsal bones," there is nothing to be said. All the evidence we possess goes to show that the position and shape of the tarsal bones is a secondary condition, and that in the large majority of cases there is no such malposition.

The third theory, that of abnormal muscular action, is also a wild one, for its supporters did not include a spasmodic contraction of certain muscles, secondary to some nerve lesion or irritation, but simply the development of some muscles out of proportion to others. Dissection shows that there is nothing to support this.

In favour of the next theory, that the deformity is due to a disordered condition of the cerebro-spinal system producing spasmodic contraction of the muscles supplied by the affected nerves, there is a good deal to be said. And, first, is there any reason to suppose that such a disordered condition does or can exist? We know that the fœtus is liable to be attacked by certain febrile affections, such as small-pox and scarlet fever, children having been born with incontestable evidence of having suffered from these; and we also know that these affections produce convulsions in young children, so we may fairly infer that the fœtus in utero may also so suffer. An article published in the Brit. and For. Med. Chi. Review records the fact that in four successive pregnancies the fœtuses died about the eighth month, and the sensations of the mother immediately preceding their deaths indicated the occurrence of convulsions. Of this lady's family nine out of ten brothers and sisters had suffered from convulsions during infancy, and six had died of the attacks. In another case in which convulsions were suspected before birth, the child was born with general muscular contractions, convulsions occurred at the time of birth, and continued until death a few days later. This case is recorded in the Edin. Med. Journal (Nov., 62). Again, diseases of the nervous system are known to be hereditary, and very young children may develop those diseases, so it is fair to infer that the fœtus may be attacked in utero. Dr. Little states as an additional argument in favour of the same theory that non-congenital club-foot occurring early in life presents the same inflexible and essential character as the congenital affection, that it advances to nearly the same grade of deformity, and is remediable by the same means, and, therefore, it is probable that both arise from similar causes. It is also a significant fact that many fœtuses with defects in the cerebro-spinal system, such as spina-bifida, and micro-cephalic fœtuses are born with club-foot, for, of course, these are the very conditions that would give rise to storms of disordered nervous action.

On the other hand, it may be urged, as Mr. Adams points out, that children born with club-foot, or feet, are frequently—in fact, generally—well nourished children, and the substance of the muscles healthy, so that when, by treatment, the foot is restored to its normal condition, the muscles perform their functions in perfect obedience to the will, and that as a rule there is no evidence of spasm in the muscles at the time of birth. However, after carefully considering the evidence for and against this theory, it must be admitted that the evidence is greatly in favour of it.

The fifth theory, that some forms are grades in the natural development of the feet which from some cause or another has been arrested, is sufficiently dealt with when it is pointed out that although this might be so in the case of varus, yet it would not explain either talipes valgus or equinus, and it is fanciful and unpractical to conclude that a cause, differing so widely from any that could produce the other forms of club-foot, should be responsible for this particular form.

The last and most fanciful theory of all is that which attributes club-foot in common with other deformities to maternal impressions; the particular deformity being determined by the nature of the idea impressed on the mother's mind during pregnancy. Although I have placed this last, it is one of, if not actually, the oldest explanations, and has held its ground with many men, and even surgeons, up to the present time, in spite of the following facts which have from time to time been put forward and proved. (*a*) From the nature of the deformity we may usually know that the error in formation must have occurred at a much earlier period than the date of the supposed cause. This is not so strikingly evident in club-foot as in some of those monstrosities where the error is one of excess. (*b*) The ovum, as soon as it leaves the ovary, ceases to be directly connected with the mother, either by the nervous or vascular systems, and is, therefore, unlikely to be affected by impressions on her mind, except in so far as its general nutrition and development is concerned. For instance, it is quite possible and even probable that a severe illness or great shock, mental or bodily, to the mother, may so affect the nutrition of the fœtus that its development is impeded, but this is a very different thing to believing that the sight of some repulsive object can cause an actual anatomical alteration in the fœtus, and even the addition of a supernumerary finger or toe. It would be interesting to ask a believer in maternal impressions as an explanation of club-foot, how he would account for the existence of similar deformities in the offspring of oviparous animals, for they do undoubtedly occur in these, and may be seen in our museums, although no possible connection can be traced between mother and offspring. (*c*) Probably there are very few women about to become mothers for the first time who do not entertain doubts and fears with regard to the condition of their offspring, but when the child is born in a healthy and well-formed condition their apprehensions are soon forgotten; and it is only in the occasional cases of deformity that the mind is ransacked for something that could have caused it, and the previous fears magnified into important facts. Mr. Noble Smith, whose remarks on this point are very convincing, suggests that anyone who wishes to convince himself on this subject should question each woman before delivery as to any impressions she may have formed upon the subject, and compare these with the actual condition of the child when born.

These are, as far as I am aware, the principal theories which have been advanced to account for congenital club-foot, and to my mind the only one which will embrace and explain the great majority of the cases one meets with, is that of disordered nervous action. Of course, there are deformities of the feet which might be included in the term club-foot which cannot be explained in this way, and which may occur in several generations of the same family, being no doubt due to an original defect in the germ—male or female—which is passed on from generation to generation, and these cases are associated with an actual error in the development of some part of the tarsus.

**Acquired Club-foot.**—The mode of origin of acquired club-foot is, of course, not nearly so obscure, and the different causes may be classified thus: (1) Disease or error in the exciting cause of muscular action, leading to either (*a*) excess of action—spasm or (*b*) deficiency of action—paralysis. (2) Mechanical impediments, such as contraction of skin, fasciæ or ligaments—of course, primary, and not following that of muscles; *e.g.*, long-continued retention in one position from pressure of bed-clothes during a long illness. (3) Deficient support—relaxation of fasciæ and ligaments. Practically we may disregard the two latter, for they only constitute a very small minority of the cases we have to treat, and confine our attention to the first, namely, disease or error in the exciting cause of muscular action. This diseased process may arise in the brain, spinal cord, or peripheral nerves. If the lesion is a cerebral one, the club-foot is only a part of an extensive deformity, which may be of the spasmodic or paralytic variety, an example of the former being found in those cases of general and long-continued convulsions occasionally occurring in newly-born children, and which have been found in some fatal cases to be accompanied by atrophy of a portion of the cerebral cortex. An example of the paralytic variety is seen in some cases of hemiplegia, though here the actual cause of the deformity is a muscular rigidity consequent on certain degenerative changes in the brain and cord. When the lesion is spinal the lower extremities only are affected, as a rule both. Of those which result in a spasmodic condition of the muscles, a familiar example is spastic paraplegia, due to sclerosis of the lateral columns or "Erle's paralysis." In this condition the muscles are equally affected, but the extensors of the foot, being stronger than the flexors, gradually attain the ascendency and talipes equinus or equino varus is the usual, though not invariable, result. The commonest form of paralytic talipes due to spinal mischief is that which results from acute anterior polio-myelitis, or, when occurring in infants, infantile paralysis, and this differs from the spasmodic variety, in that certain muscles or groups of muscles are picked out so that we may get any form of talipes following it, on account of the loss of balance between the different groups, which results in a gradual shortening of the unopposed muscles, and a gradual lengthening of those paralysed. Should all the muscles below the knee be attacked at once the usual result is equino-varus, that being the natural position into which the foot falls in a recumbent position, and to which the ligaments, fasciæ, &c, gradually accommodate themselves. It is to this class that by far the largest number of acquired talipes cases are to be referred. Lesions of the peripheral nerves are by no means a common cause of talipes, but by analogy with other parts of the body we may infer that they are a possible cause—for instance, the contraction that follows old facial paralysis. Before concluding the subject of causation perhaps I should mention various forms of reflex irritation as a possible explanation of some of the spasmodic cases, but I would not lay much stress on this explanation.

